# Oxidative damage within alternative DNA structures results in aberrant mutagenic processing

**DOI:** 10.1093/nar/gkaf066

**Published:** 2025-02-14

**Authors:** Maha Zewail-Foote, Imee M A del Mundo, Alex W Klattenhoff, Karen M Vasquez

**Affiliations:** Department of Chemistry and Biochemistry, Southwestern University, 1001 E University Ave, Georgetown, TX 78626, United States; Division of Pharmacology and Toxicology, College of Pharmacy, The University of Texas at Austin, Dell Pediatric Research Institute, 1400 Barbara Jordan Blvd. Austin, TX 78723, United States; Division of Pharmacology and Toxicology, College of Pharmacy, The University of Texas at Austin, Dell Pediatric Research Institute, 1400 Barbara Jordan Blvd. Austin, TX 78723, United States; Division of Pharmacology and Toxicology, College of Pharmacy, The University of Texas at Austin, Dell Pediatric Research Institute, 1400 Barbara Jordan Blvd. Austin, TX 78723, United States

## Abstract

Genetic instability is a hallmark of cancer, and mutation hotspots in human cancer genomes co-localize with alternative DNA structure-forming sequences (e.g. H-DNA), implicating them in cancer etiology. H-DNA has been shown to stimulate genetic instability in mammals. Here, we demonstrate a new paradigm of genetic instability, where a cancer-associated H-DNA-forming sequence accumulates more oxidative lesions than B-DNA under conditions of oxidative stress (OS), often found in tumor microenvironments. We show that OS results in destabilization of the H-DNA structure and attenuates the fold increase in H-DNA-induced mutations over control B-DNA in mammalian cells. Furthermore, the mutation spectra revealed that the damaged H-DNA-containing region was processed differently compared to H-DNA in the absence of oxidative damage in mammalian cells. The oxidatively modified H-DNA elicits differential recruitment of DNA repair proteins from both the base excision repair and nucleotide excision repair mechanisms. Altogether, these results suggest a new model of genetic instability whereby H-DNA-forming regions are hotspots for DNA damage in oxidative microenvironments, resulting in its altered mutagenic processing. Our findings provide valuable insights into the role of OS in DNA structure-induced genetic instability and may establish H-DNA-forming sequences as promising genomic biomarkers and potential therapeutic targets for genetic diseases.

## Graphical Abstract

**Graphical Abstract:**
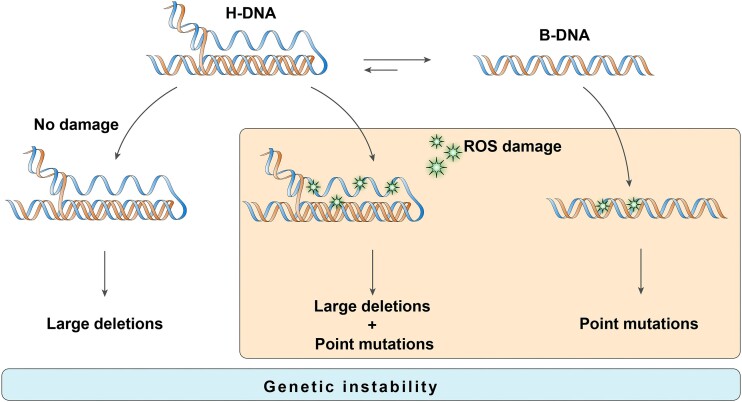

## Introduction

Genetic instability, a hallmark of cancer and other genetic diseases, can result from endogenous and exogenous sources of DNA damage. For example, cellular reactive oxygen species (ROS) can cause oxidative DNA damage from both endogenous [e.g. aerobic metabolism, oxidative stress (OS)] and exogenous sources such as environmental genotoxic chemicals and ultraviolet (UV) light. In particular, cancer cells have been shown to exhibit elevated levels of ROS compared to normal cells due to their susceptibility to OS [1, 2]. The genome is a major target of ROS-mediated damage, contributing to epigenetic modifications, mutations, and human carcinogenesis [3]. In particular, ROS leads to the formation of a variety of base oxidation products formed at both purines and pyrimidines. Among the most common ROS-induced oxidative lesion is 8-oxo-7,8-dihydro-2′-deoxyguanosine (8-oxodG), which is estimated to occur at a level of up to 10^5^ lesions per cell per day in cancer tissues [4]. Hence, 8-oxodG can serve as a ubiquitous marker of OS [5]. 8-oxodG is also a pre-mutagenic oxidative lesion and can result in mutations, predominantly G:C to T:A transversions, if left unrepaired or repaired in an error-generating fashion [5, 6]. Accumulation of mutagenic ROS-induced lesions is a threat to genome integrity and has been shown to be associated with age-related diseases, such as Alzheimer’s, Parkinson’s, Huntington’s disease [7–9], and cancer [10, 11].

In addition to ROS, alternative DNA structures (i.e. non-B DNA) can serve as endogenous sources of genetic instability [12–17]. Sequences with the capacity to adopt non-B DNA structures (e.g. H-DNA) are abundant in the human genome and enriched at specific regions, including gene regulatory regions and hotspots of genetic instability [18–20]. Of clinical relevance, we have found that non-B DNA-forming sequences are enriched at translocation and mutation hotspots in human cancer genomes, and can stimulate the formation of DNA double-strand breaks via replication-dependent and replication-independent mechanisms [17,20–24]. Non-B DNA can be recognized and processed by DNA repair proteins. For example, H-DNA-forming sequences are processed in a mutagenic fashion by the nucleotide excision repair (NER) mechanism, leading predominantly to large deletions [23], whereas proteins from the NER and mismatch repair (MMR) pathways are involved in Z-DNA-induced genetic instability [24].

Guanine-rich sequences, many of which have the capacity to adopt non-B DNA structures, are more susceptible to oxidative DNA damage than other nucleotides given that guanine has the lowest oxidation potential among the nucleobases [25]. For example, non-B DNA structures, such as G4 DNA, have been shown to be targets for base oxidation under ROS-generating conditions, which can result in alterations in biological function [26–28]. Interestingly, growing evidence suggests a functional role of oxidized bases in controlling the formation of G4 structures, and thereby modulating epigenetic gene regulation [29, 30]. Similarly, oxidative damage within Z-DNA-forming sequences, which are enhanced in promoter regions, was found to impact gene expression [31].

**Figure 1.**
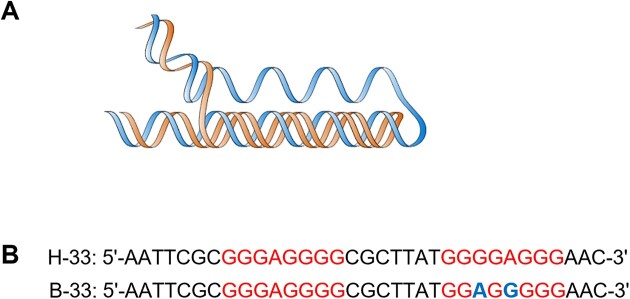
H-DNA forms at polypurine–polypyrimidine mirror repeats. (**A**) Schematic representation of an intramolecular triplex (H-DNA) structure where one strand folds back and reverse Hoogsteen hydrogen bonds to the purine-rich strand of the underlying duplex, leaving a pyrimidine-rich single-stranded region. (**B**) H-DNA forms at polypurine–polypyrimidine mirror repeats such as the H-33 sequence derived from the human c-*MYC* promoter region. B-33 is the 33-bp control B-DNA sequence that has two base substitutions that disrupt the mirror symmetry to prevent H-DNA formation [17].

The occurrence of 8-oxodG is not uniformly distributed across the genome. A collection of genome-wide mapping results have revealed that 8-oxodG-damaged sites often co-localize and accumulate at key regulatory elements, such as DNA replication origins [32] and guanine-rich promoter regions in human cells [33]. In addition, particular genomic regions are more vulnerable to oxidative DNA damage than others due to the local environment, including DNA flexibility and nucleotide sequence context, which characterize many non-B DNA structures [26–28,34–36]. Notably, several types of non-B DNA are susceptible to oxidation due to their repetitive G-rich sequences and structural arrangements [37]. Further, the loop region of hairpins have been shown to be more susceptible to base oxidation [38]. The presence of a single-stranded region in H-DNA is also likely to be more susceptible to ROS-induced DNA damage (Fig. 1A). In addition, oxidative DNA damage located within some non-B DNA structures can impair their repair processing [39]. For example, the base excision repair (BER) glycosylase, hOGG1, was found to remove 8-oxodG within a hairpin structure at a slower rate compared to duplex DNA, which could then result in an accumulation of DNA damage [39].

Overall, these studies indicate that there are regions in the human genome where oxidative DNA damage occurs more frequently and/or are repaired less efficiently in human cells. These previous studies suggest an association between oxidatively damaged regions and altered biological outcomes that can impact genetic instability. A recent study of genome-wide mapping of 8-oxodG-enriched promoters in human epithelial cells found that these sites were associated with translocation breakpoints in breast cancer cells and showed characteristic features of genetic instability [33]. Further, we have shown that 8-oxodG was significantly enriched in several non-B DNA-forming sequences, including H-DNA, showing co-localization between DNA structure-forming motifs and regions of 8-oxodG lesions [40].

Given the overlap between oxidatively damaged regions and non-B DNA-forming sequences, there may be an association between this correlation and increased genetic instability. The complex interplay between oxidative lesions and non-B DNA-induced genetic instability has not been well studied. To gain better insight into this multifaceted interaction, we sought to examine how oxidative DNA lesions may modulate H-DNA-induced genetic instability. Here, we provide the *first* description of the impact of oxidative lesions on the mutagenic potential and processing of H-DNA in mammalian cells through structural and functional characterization. We posit that H-DNA structures are more susceptible to oxidative damage and that oxidative lesions are processed differently within H-DNA compared to canonical B-DNA. In particular, the increased susceptibility may arise from the unique structural features of H-DNA, which forms at mirror repeats that are rich in guanines (Fig. 1B). H-DNA consists of an intramolecular triplex (where the third strand folds back and binds the duplex through reverse Hoogsteen hydrogen bonding), an exposed single-stranded region, and loop (Fig. 1A). We found that an H-DNA-forming sequence from a translocation breakpoint hotspot in the c-*MYC* gene in Burkitt lymphoma accumulated higher levels of 8-oxodG lesions than canonical B-DNA-forming sequences. Furthermore, increasing levels of oxidative damage attenuated the fold increase of H-DNA-induced mutation frequencies over control B-DNA in mammalian cells. Based on the mutation spectra and protein–DNA interactions, oxidatively damaged H-DNA-forming sequences are processed differently in cells compared to H-DNA in the absence of oxidative damage. Our analyses provide insight into the molecular mechanisms involved in H-DNA processing in the presence of oxidative DNA damage and provide a novel paradigm for the increased genetic instability at these hotspot regions.

## Materials and methods

### Construction and preparation of substrates for the immuno-slot blot assay

To prepare the substrates for the slot blot assay, 2,100 bp plasmids containing the H-DNA-forming sequence (H-33: 5′-AATTCGCGGGAGGGGCGCTTATGGGGAGGGAAC) or control B-DNA sequence (B-33: 5′-AATTCGCGGGAGGGGCGCTTATGGAGGGGGAAC) were constructed from the previously described 5,100 bp parent pMycY and pMycAG plasmids [17]. pMycY and pMycAG were digested with restriction enzymes HpaI and PvuII at 37°C and the resulting fragments were separated by agarose gel electrophoresis. The 2,100 bp fragment was excised from the gel, purified using the QIAquick gel extraction kit (QIAGEN), and then incubated with blunt/TA ligase master mix (New England Biolabs) at room temperature for 15 min. *Escherichia coli* cells were transformed with the purified 2,100 bp plasmid and isolation of plasmid DNA was performed using a Plasmid Maxi Kit (QIAGEN). DNA concentrations were measured spectrophotometrically using a Nanodrop and the formation of H-DNA was verified using an S1 nuclease assay as described [17].

### Induction of ROS-induced oxidative damage

To induce varying levels of OS, supercoiled plasmid DNA was incubated with 50 μM hydrogen peroxide and 3 or 10 μM of ferrous sulfate in 30 mM sodium acetate (pH 5.2), 25 mM NaCl, and 4 mM MgCl_2_. Reactions were incubated for 30 min at room temperature. Stock solutions of ferrous sulfate and hydrogen peroxide were prepared immediately prior to use.

### Purification of the 33 bp B-DNA (B-33) or H-DNA (H-33) fragments

Oxidatively damaged or undamaged 2,100 bp plasmids (5 μg) were double digested with restriction enzymes EcoRI-HF and XhoI (New England Biolabs) in rCutSmart buffer at 37°C to yield a 33-bp fragment containing the H- or control B-DNA-forming sequence as well as the large EcoRI-XhoI fragment. The resulting 33-bp fragment was isolated using a modified protocol from the QIAquick PCR Purification Kit (QIAGEN). Five volumes (250 μl) of Buffer PB were mixed with the 50 μl digestion reactions, added to the QIAquick PCR Purification column, and spun for 1 min at 13,000 rpm. Because the QIAquick PCR Purification columns bind DNA larger than 100 bp, the eluate contained the 33-bp fragment (H-33 or B-33) while the large EcoRI-XhoI fragment was retained on the column. The eluate containing the 33-bp DNA fragment was ethanol precipitated and the pellet was resuspended in 50 μl of elution buffer. The QIAquick PCR Purification Kit protocol was followed according to the manufacturer to elute the large EcoRI-XhoI fragment from the column. The purification was confirmed by running the DNA fragments on a 1.8% agarose gel along with DNA molecular weight markers.

### Immuno-slot blot assay to assess 8-oxodG levels

The immuno-slot blot assay was carried out according to the protocol recommended by the supplier (Bio-Rad, Catalog #1706542). After transferring the purified DNA fragments, the nitrocellulose membrane was baked for 2 h at 80°C to immobilize the DNA. Membranes were blocked in tris-buffered saline containing 0.05% Tween (TBS-T) and 5% milk for 1–2 h at room temperature. The membrane was incubated with primary rabbit anti-8-oxodG antibody (Abcam, Catalog #ab48508) diluted 1:500 in TBS-T with 5% milk for 16 h at 4°C. After washing three times in TBS-T for 5 min, membranes were incubated with secondary horseradish peroxidase-conjugated goat anti-rabbit IgG (Bio-Rad #1706516, 1:2000 dilution in TBS-T) for 1 h and then washed three times for 5 min. Signals were visualized with Bio-Rad’s Clarity Western ECL chemiluminescent substrate. After chemiluminescent visualization, blots were stained with SYBR Gold (Invitrogen) to quantify the total amount of DNA immobilized per well. Blots were scanned using a gel documentation system (GelDoc XR + System) to perform chemiluminescent or UV detection. The intensities of the bands were quantified using the ImageJ software (NIH, https://imagej.net/ij/). The intensity of 8-oxodG for each sample was first adjusted to its respective SYBR Gold intensity which represents the total amount of DNA immobilized to the membrane. Sample mean and standard deviation was then calculated from four biological replicates (*n* = 4). The fold change was determined by normalizing all sample means to the undamaged B-33 control. Statistical differences were calculated using an unpaired, one-sided Wilcoxon rank-sum test (α = 0.05, GraphPad Prism software v10).

### Quantitative polymerase chain reaction to measure the accumulation of oxidized bases in the pyrimidine- and purine-rich strands of the H-DNA-forming sequence

The accumulation of oxidized lesions within the pyrimidine- and purine-rich strands of the H-DNA-forming sequence was determined using a modified quantitative polymerase chain reaction (qPCR) assay, as previously described [41]. Oxidatively damaged or undamaged 2,100 bp plasmids were linearized using PsiI-v2 (New England Biolabs) for 1 h at 37°C followed by treatment with either FPG (formamidopyrimidine [fapy]-DNA glycosylase) or EndoVIII for another 1 h in rCutSmart Buffer (New England Biolabs) to induce lesion-specific removal of oxidized bases. DNA concentrations were measured using the Qubit double-stranded DNA 1× high sensitivity quantification kit (Thermo Fisher Scientific). Glycosylase-treated or untreated templates (0.04 pg) were combined with 500 nM of either the H-DNA forward primer (5′-AGGGCGACACGGAAATGTTGAATAC) or reverse primer (5′- GAACCCCACCACAGCTCG) (Integrated DNA Technologies) in 20 μl of 1× iTaq Universal SYBR Green Supermix (Bio-Rad, Catalog #1725120) and amplified using a Bio-Rad thermocycler (95°C for 3 min, then 95°C for 30 s, and 58°C for 60 s for 20 cycles). The opposite primer was added and qPCR was performed on the amplified, glycosylase-treated or untreated templates (95°C for 3 min, then 95°C for 30 s, and 58°C for 60 s for 44 cycles), including negative no-template controls using a CFX96 Real-Time PCR System (Bio-Rad, Catalog #CFX96). Quantification cycles (Cq) were determined by CFX Maestro Software (Bio-Rad). Each sample was performed in duplicate and the Cq values were averaged to technical means (Cq_S_). Fold increase was then calculated for each sample over the Cq of the reference amplicon template, the undamaged template without treatment of lesion-specific glycosylases (Cq_C_) using the equation 2^(Cq_S_ − Cq_C_). Biological sample means and standard deviations were then calculated from three biological replicates (*n* = 3). Statistical differences were calculated using an unpaired, one-sided Welch’s *t*-test (α = 0.05, GraphPad Prism v10).

### Mutagenesis assays

The mutagenesis assay was performed as we have previously described [17]. Mammalian COS-7 cells were transfected with oxidatively damaged or undamaged reporter plasmids (3 μg) containing the H-DNA-forming sequence from the human *c-MYC* gene promoter (pMycY) or a control B-DNA-forming sequence (pMycAG) using GenePORTER (AMSBIO) [17]. Plasmid DNA was recovered after 48 h and isolated using the QIAprep Spin Miniprep kit (QIAGEN). Isolated plasmids were digested with Dpn1 (New England Biolabs) to remove unreplicated DNA, followed by phenol/chloroform extraction and ethanol precipitation. Pellets were resuspended in 10 μl of nuclease-free water and 1 μl of the plasmid preparation was used to transform electrocompetent *E. coli* strain MBM7070. Mutations in the *supF* gene were monitored by blue–white screening on LB (Luria-Bertani)-carbenicillin agar plates containing X-Gal and IPTG (Isopropyl β-D-1-thiogalactopyranoside). Mutation frequencies were calculated as the number of white mutant colonies divided by the total number of colonies (blue + white) counted. Experiments were repeated at least three times and at least 50,000 total colonies were counted per experiment. For the mutation spectra, between 7 and 30 randomly selected mutant colonies from each group was sequenced by Sanger sequencing.

### Thermal analysis and circular dichroism spectroscopy

Oligonucleotides were purchased from Midland Certified Reagent Co. (Midland, TX). Sequences can be found in Supplementary Fig. S1. Oligonucleotides (11 μM) were annealed in triplex-forming buffer [20 mM sodium cacodylate, pH 7.0, 100 mM NaCl, 0.1 mM ethylenediaminetetraacetic acid (EDTA), and 10 mM MgCl_2_] by heating to 95°C for 5 min followed by gradually cooling to room temperature. UV absorbance was monitored as a function of temperature at a rate of 0.4°C/min at 260 nm on a Cary 100 UV-Vis spectrophotometer equipped with a Peltier temperature controller (Agilent Technologies, Santa Clara, CA). Melting temperatures (T_m_) were calculated by taking the first derivative of the melting curve and averaged over at least three independent experiments.

The circular dichroism (CD) spectra of annealed modified or unmodified R2 (10 μM in 300 μl) in 0.1 cm quartz cuvettes were recorded at 25°C using a Jasco J-815 (Jasco Inc., Easton, MD). Spectra were collected between 200 and 350 nm and scanned three times at a rate of 100 nm/min with a two second response time. CD spectra are representative of three independent experiments.

### Gel mobility assays

Annealed modified or unmodified R2 oligonucleotides were run on a 20% native polyacrylamide gel (20 mM sodium cacodylate, 100 mM NaCl, 0.1 mM EDTA, 10 mM MgCl_2_) in Tris-borate-magnesium (TBM) buffer (0.4 M Tris, 0.4 M boric acid, 10 mM MgCl_2_) at 45 volts and post-stained with SYBR Gold (ThermoFisher Scientific) as previously described [42].

### Chromatin immunoprecipitation assay

Chromatin immunoprecipitation (ChIP) assays were carried out using a modified protocol of the SimpleChIP Enzymatic Chromatin IP Kit (Cell Signaling, Catalog #9003). All reagents were used from this kit unless otherwise noted. Briefly, undamaged or oxidatively damaged pMyc plasmids were transfected into ∼8 × 10^6^ U2OS cells (ATCC, Catalog #HTB-96) using GenePORTER 2 (AMSBio, Catalog #T202015). After 24 h, cells were fixed and lysed to collect nuclei that were processed into fragmented chromatin and stored at −80°C until needed. Immunoprecipitations were prepared for each sample by aliquoting the fragmented chromatin into ChIP Buffer containing the negative control Normal Rabbit IgG (DF 1/500, Cell Signaling, Catalog #2729), anti-APE1 (DF 1/200, Proteintech, Catalog #10203), anti-XPA (DF 1/200, Abcam, #85914), or an input loading control set aside until elution. Immunoprecipitations were complexed overnight at 4°C with rotation, precipitated and washed with magnetic Protein A/G beads, eluted, and processed to isolate sample DNA that was associated with the respective target protein.

Sample DNA was loaded in triplicate into a 96-well plate with iTaq Universal SYBRGreen Supermix (Bio-Rad, Catalog #1725120) alongside its associated negative control and input control for each biological replicate. qPCR of the reporter sequence was conducted using a CFX96 Real-Time PCR System (Bio-Rad, Catalog #CFX96) and associated software to determine percent input (% Input) for each sample. Sample Cq values were first averaged to calculate Cq technical means. Each sample Cq technical mean was then adjusted to its input loading control Cq technical mean and expressed as a percent of input (% Input). Biological sample % Input means and standard deviations were then calculated from four biological replicates (*n* = 4). Statistical differences were finally calculated using an unpaired, one-sided Wilcoxon rank-sum test (α = 0.05, GraphPad Prism v10).

## Results

### Strand-specific accumulation of oxidative damage in H-DNA is greater than that in B-DNA

**Figure 2.**
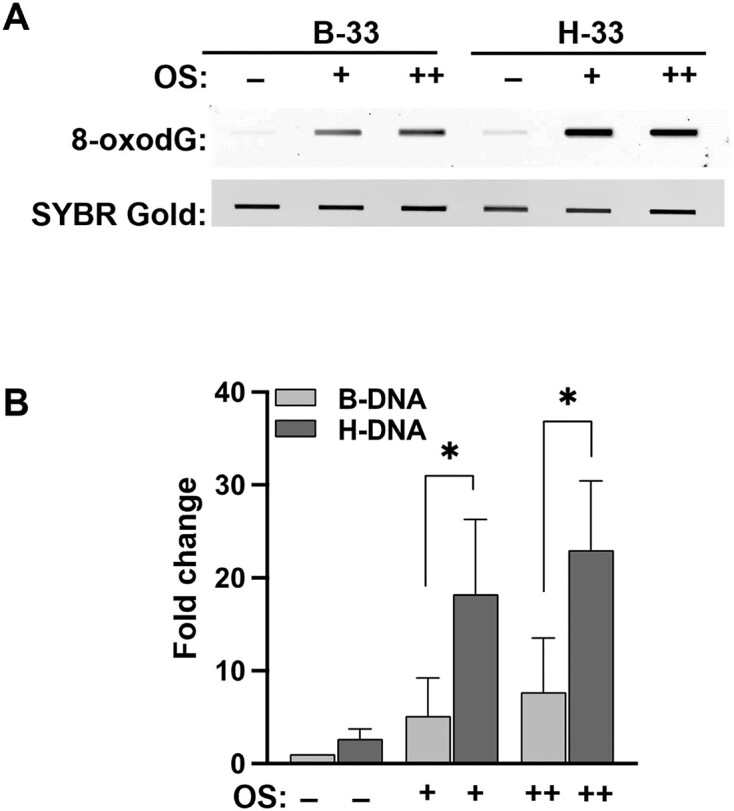
Oxidative damage accumulates in H-DNA over control B-DNA. (**A**) Representative slot blot of the untreated or oxidatively damaged H-33 and B-33 fragments containing either the H-DNA-forming sequence or control B-DNA sequence, respectively, under varying conditions of OS. (**B**) Quantification of the slot blot as shown in panel (**A**). Intensities of 8-oxodG were adjusted to the total amount of DNA bound on the membrane and normalized to the control untreated B-33 to show the fold increase. 8-oxodG is significantly higher in H-33 compared to B-33 under oxidizing conditions (*n* = 3).

DNA oxidation by ROS is dependent on the local sequence content and secondary structure, ultimately leading to a heterogeneous distribution of DNA damage within the genome [43]. Accumulation of oxidative damage in particular genomic regions may be associated with non-B DNA-forming sequences. Here, we evaluate if H-DNA, which contains an exposed single-stranded region, is a hotspot for oxidative DNA damage. To evaluate the extent to which H-DNA accumulates oxidative damage relative to B-DNA, we first performed an immuno-slot blot assay (Fig. 2A) using a substrate with an H-DNA-forming sequence from the human *c-MYC* promoter that maps to a translocation breakpoint hotspot in Burkitt lymphoma (Fig. 1B) [42]. The levels of the ubiquitous guanine oxidation product, 8-oxodG, which is a common biomarker of OS-induced DNA damage, were measured within a 33-bp fragment from the substrate containing the H-DNA- or a control B-DNA-forming sequence, H-33 and B-33, respectively. Both fragments contain the same guanine and cytosine (GC) content, but the B-DNA-forming fragment (B-33) cannot form the H-DNA structure due to two base substitutions that disrupt the homopurine–homopyrimidine mirror repeat required for H-DNA structure formation [17]. DNA reporter substrates containing the B-33 or H-33 fragments were prepared and purified using standard alkaline lysis methodology. While this preparation method has the potential to induce non-B DNA structure formation, the S1 nuclease sensitivity experiments confirmed the presence of H-DNA structures in the H-33 substrates (Supplementary Fig. S2). To directly measure the relative damage levels within the H-33 and B-33 sequences, the DNA reporters containing either the H-33 or B-33 DNA fragments were subjected to OS mediated by the Fenton reaction to induce ROS-mediated DNA damage *in vitro*. The undamaged and damaged reporters were then digested with two restriction enzymes specific for the regions surrounding the H-DNA- and B-DNA-forming sequences. The resulting H-33 or B-33 fragments were isolated, immobilized onto a nitrocellulose membrane, and probed with an anti-8-oxodG antibody followed by imaging (Fig. 2A). Chemiluminescent intensity was then quantified as described in the ‘Materials and methods’ section (Fig. 2B). A small amount of background 8-oxodG was detected in samples not exposed to oxidizing conditions (Fig. 2). As expected, the levels of 8-oxodG were elevated with increased levels of OS. Interestingly, a significant increase in 8-oxodG was observed within the H-DNA-containing H-33 fragment compared to the control B-DNA fragment of the same length and GC content (Fig. 2B). This result supports our hypothesis that H-DNA is a hotspot for oxidative damage accumulation presumably due to its structural alterations.

**Figure 3.**
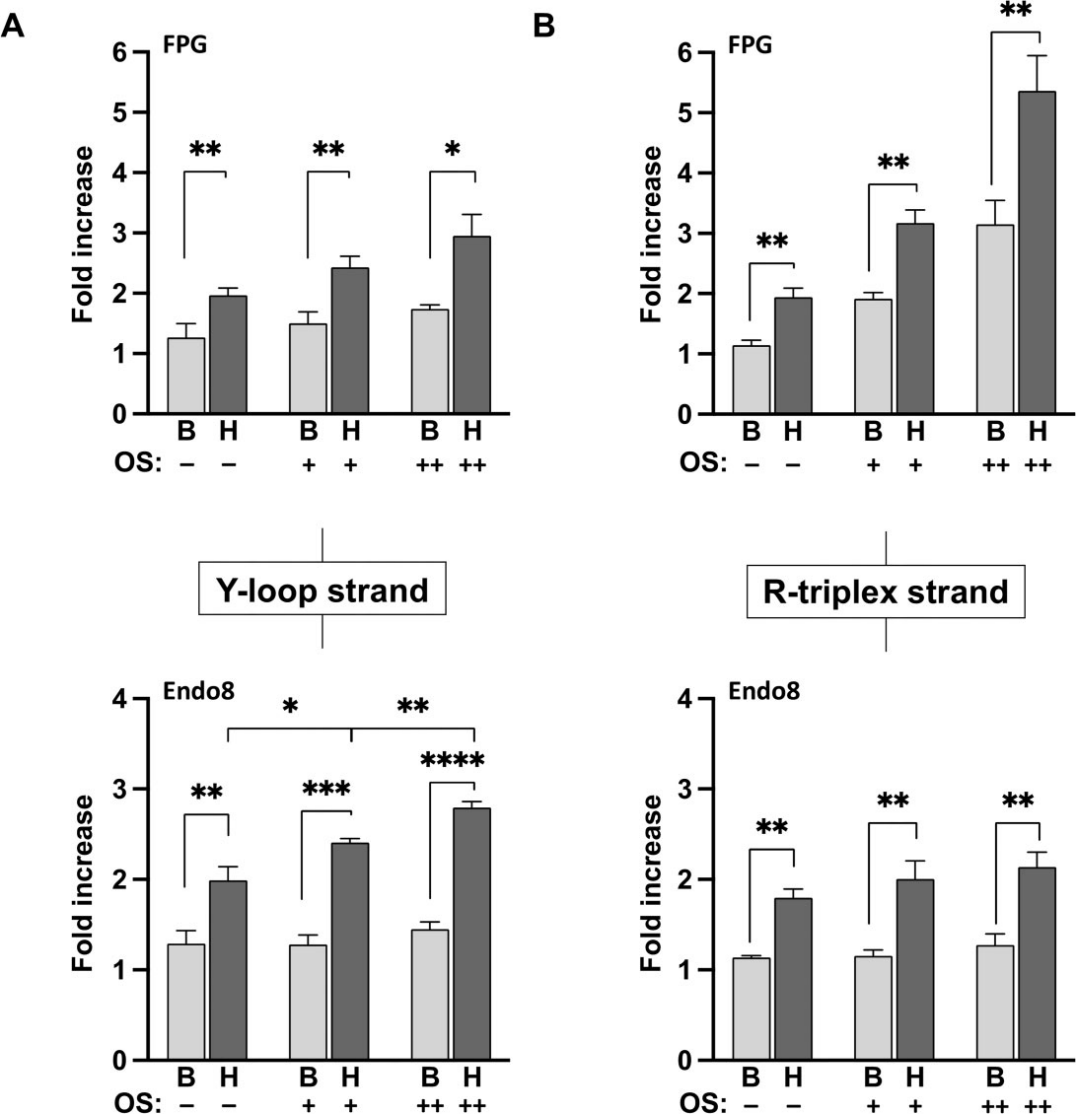
Distribution of oxidative damage in H-DNA is strand-specific. FPG- and Endo8-sensitive lesions formed within the Y-loop strand (**A**) or the R-triplex strand (**B**) of the H-DNA-forming sequence compared to the control B-DNA sequence. Quantitative PCR (qPCR) was used to measure the fold increase in Cq values between oxidatively damaged templates that were FPG- or Endo8-treated compared to undamaged B-DNA without treatment with a damage-specific glycosylase. Results represent the mean ± standard deviation (SD) from at least three independent experiments, *P < .05, **P < .01 ***P < .001, and ****P < .0001.

We next utilized qPCR to further evaluate the extent to which H-DNA accumulates OS-induced damage relative to B-DNA on individual DNA strands in the region containing the H-DNA-forming sequence. Specifically, the strand-specific accumulation of oxidized purine and pyrimidine lesions within the purine-rich intramolecular triplex (R-triplex) strand or the pyrimidine-rich, single-stranded loop (Y-loop) strand of H-DNA were quantified relative to the undamaged control B-DNA template. For qPCR analysis, undamaged and damaged DNA reporters used in the slot blot assay were linearized with a restriction enzyme to remove secondary and tertiary structures which could interfere with PCR amplification, followed by treatment with either FPG (specific to oxidized purine bases including 8-oxodG) or Endonuclease VIII (Endo8, specific to oxidized pyrimidine bases). FPG and Endo8 excise the damaged bases and cleave the DNA backbone to create a one nucleotide base gap, which inhibits Taq polymerase elongation during qPCR [41]. As a result, templates containing oxidative damage within the amplified region are not as efficiently amplified compared to the undigested, undamaged templates, allowing for relative quantification of oxidative DNA damage levels resulting from the loss in fluorescence signal. To specifically detect oxidized purine or pyrimidine bases on the R-triplex strand or the Y-loop strand, a first round of linear PCR amplification was used with only the forward or reverse primer to enrich for one of the DNA strands within H-DNA, followed by a second round of qPCR with the opposite primer added. Comparing fold increases above undamaged, undigested B-DNA templates, H-DNA templates treated with FPG have significantly more oxidized purine bases than B-DNA templates at all levels of OS, even in undamaged samples (Fig. 3). Notably, increases in OS-induced oxidized purine lesion formation was more pronounced in the R-triplex strand compared to the Y-loop strand. Similarly, H-DNA templates have more oxidized pyrimidine bases than B-DNA templates in all Endo8-treated samples. Strikingly, OS-induced oxidized pyrimidine lesion formation increased by almost 20% within the Y-loop of H-DNA but not in the B-DNA templates (Fig. 3A). These findings suggest that H-DNA accumulates more DNA damage from OS than B-DNA, especially within the single-stranded, pyrimidine-rich Y-loop of H-DNA, as we predicted.

### Oxidative lesions destabilize the H-DNA structure

**Figure 4.**
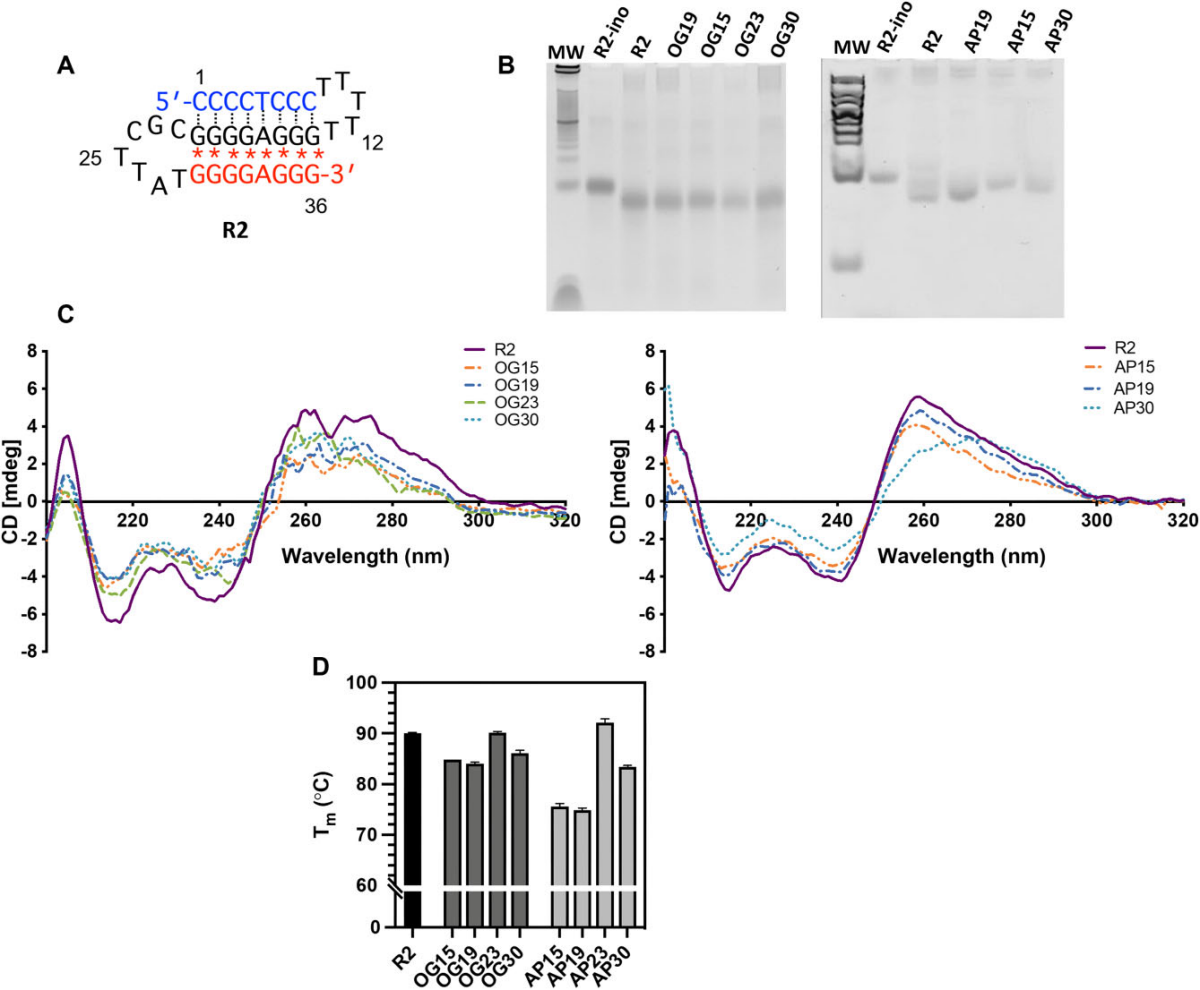
Impact of oxidative lesions on H-DNA formation and stability. (**A**) Specific guanine bases were replaced with either 8-oxodG (OG) or an abasic site (AP) based on the numbering scheme shown in the 36-bp H-DNA-forming R2 substrate. Guanine substitutions were placed either in the Watson–Crick duplex region (positions 15 and 19), the single-stranded loop (position 23), or within the triplex reverse Hoogsteen strand (position 30); *Reverse Hoogsteen hydrogen bonds. (**B**) Native polyacrylamide gels showing mobilities of H-DNA-forming R2 compared to lesion-modified R2 oligonucleotides containing a single OG or AP at positions 15, 19, 23, or 30 within the H-DNA substrate. R2-ino is a non-H-DNA-forming sequence where inosines replace guanines on the third strand resulting in hairpin formation due to the loss of the exocyclic amino groups. (**C**) Representative CD spectra from three independent trials of R2, OG-, or AP-modified R2 sequences. (**D**) Melting temperatures (T_m_) of R2, OG-, or AP-modified R2 sequences. T_m_ is reported as an average value ± SD from at least three independent experiments.

Since we demonstrated that H-DNA accumulates more DNA damage from OS than B-DNA, we next speculated that oxidation may affect the stability of the H-DNA structure. To investigate, we evaluated the impact of two major lesions resulting from oxidation, 8-oxodG and abasic sites, on H-DNA structure formation and stability. Abasic sites are also intermediates of 8-oxoG processing by BER [44]. We utilized our previously characterized intramolecular foldback H-DNA-forming substrate (R2) [42] and replaced specific guanines with either an 8-oxodG or an abasic site at varying positions (Fig. 4A and Supplementary Fig. S1). These substitutions included guanines located in the Watson–Crick duplex region (positions 15 and 19), the single-stranded loop (position 23), and the reverse Hoogsteen hydrogen-bonded triplex region (position 30) (Fig. 4A and Supplementary Fig. S1). These guanines have the potential to be more susceptible to oxidative damage [38,45–47].

Native polyacrylamide gel electrophoresis (PAGE) and CD spectroscopy were used to determine if these oligonucleotides containing a single 8-oxodG or an abasic site are still capable of forming H-DNA structures, as previously described for this sequence [42]. The gel migration patterns of the modified H-DNA substrates containing a single 8-oxodG at positions 15, 19, 23, and 30 (OG15, OG19, OG23, and OG30, respectively) are similar to that of the unmodified H-DNA substrate R2, indicating that the H-DNA structure still forms when an 8-oxodG is incorporated at these locations (Fig. 4B). When an abasic site is positioned within the four consecutive guanines in the Watson–Crick duplex region, AP19, the migration pattern was also similar to R2. However, when an abasic site is located within the Watson–Crick duplex region at position 15 (AP15) or the reverse Hoogsteen hydrogen-bonded triplex region at position 30 (AP30), the mobilities of the modified substrates are slower than R2, suggesting structure destabilization leading to a less compact folded structure. In the control R2-ino oligonucleotide, the guanines in the third strand are replaced with inosines which lack the exocyclic 2-amino group required for reverse Hoogsteen H-bonding, preventing H-DNA/triplex formation. Thus, R2-ino can only form a hairpin structure, and shows a slower gel migration pattern (Fig. 4B). The different gel migration patterns of AP15 and AP30, which are between R2 and R2-ino suggest a conformational polymorphism induced by the oxidative lesion at these specific locations.

CD spectroscopy was also employed to evaluate the effect of incorporating a single 8-oxodG lesion or an abasic site on the H-DNA/triplex structure. The appearance of a negative peak at ∼210–220 nm is characteristic of triplex DNA formation [48]. Based on the CD spectra, the lesion-modified R2 sequences exhibit the characteristic triplex spectral peak regardless of lesion type and location within R2 (Fig. 4C). These modified sequences also exhibit smaller changes in CD intensity at 210–220 nm compared to R2 which may be indicative of subtle structural differences or the formation of less stable intramolecular triplexes. In addition, a CD shift from 260 to 275 nm for AP30 suggests structural deviations from R2. Based on the native PAGE and CD spectroscopy results, R2 sequences containing either a single 8-oxodG or an abasic site still have the capacity to form H-DNA/triplex structures.

Next, we explored the impact of a single 8-oxodG or an abasic site on the thermal stability of the H-DNA secondary structure by measuring the melting temperatures (T_m_) of the R2 sequences containing site-specific base modifications. The absorbance at 260 nm was monitored as a function of increasing temperature for both unmodified and site-specifically modified R2 substrates. The unmodified R2 is a markedly stable structure with a T_m_ of ∼90°C (Fig. 4D) under our experimental conditions. The presence of a single 8-oxodG base at positions 15, 19, or 30 (OG15, OG19, or OG30) exhibited a destabilizing effect as demonstrated by as much as an ∼6°C reduction in T_m_ compared to R2 (Fig. 4D). On the other hand, a single 8-oxodG located within the H-DNA loop (OG23) does not alter the T_m_. The presence of an abasic site, however, destabilized the H-DNA structure to a greater extent compared to both the unmodified R2 and the 8-oxodG-modified substrates in the same locations with a decrease in T_m_ of as much as 15°C. Similar to OG23, there was no substantial change in T_m_ when an abasic site was located within the loop (AP23). Hence, we did not further examine the effect of an abasic site at this position on H-DNA structure formation using CD or gel electrophoresis as OG23 showed no structural changes compared to R2. The change in T_m_ also depends on the location of the 8-oxodG and abasic site within the H-DNA structure with lesions positioned on the Watson–Crick strand (positions 15 and 19) being more destabilizing than a lesion positioned within the reverse Hoogsteen hydrogen-bonded triplex region (position 30) (Fig. 4D and Supplementary Fig. S1). The H-DNA structure destabilization associated with the lower T_m_s can be ascribed to the altered H-bonding in the triplex region (8-oxodG) and the absence of the stabilizing H-bonding interactions caused by the removal of the base (abasic site). Together, these results indicate that the R2 sequences containing a single 8-oxodG or an abasic site modification are still capable of forming H-DNA structures as indicated by the CD spectra and PAGE gels; however, the lesions reduce the overall stability of the H-DNA structure as assessed by T_m_ measurements, depending on the location of the lesion.

### Oxidative damage attenuates H-DNA-induced mutation frequencies over control B-DNA in mammalian cells

**Figure 5.**
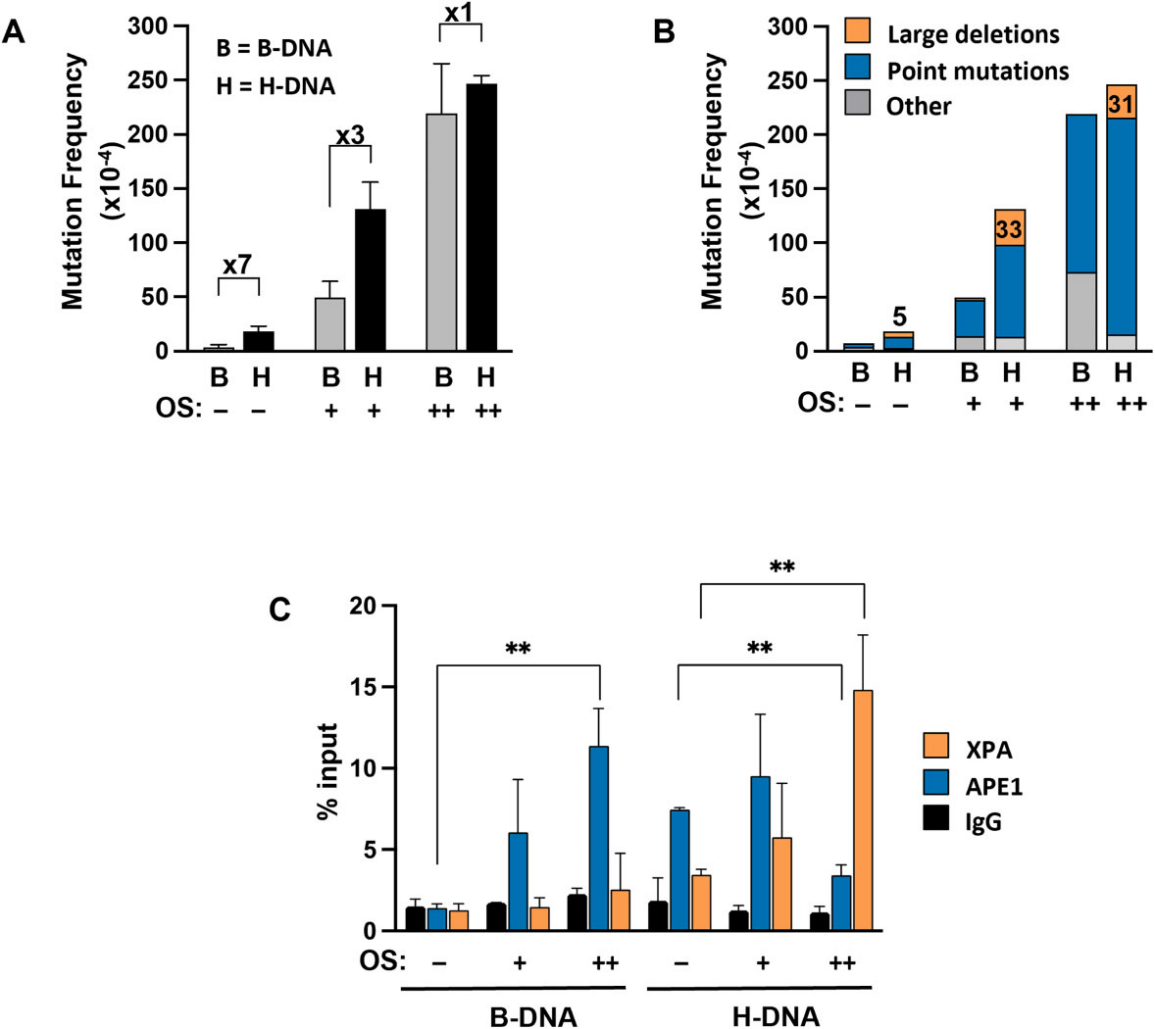
Oxidative damage attenuates the fold increase of H-DNA-induced mutation frequencies over B-DNA leading to differential enrichment of APE1 and XPA. (**A**) Mutation frequencies of mutation reporters containing H-DNA- (‘H’) or B-DNA-forming sequences (‘B’) under conditions of OS. (**B**) Mutation spectra of the mutant colonies from each experimental group in panel (A). Mutations were grouped as either large deletions, point mutations, or other types of mutations. The prevalence of large deletions increased from 5 to ∼30 per 10,000 total colonies from untreated H-DNA to oxidatively damaged H-DNA as shown. Experiments were repeated at least three times and at least 50,000 total colonies were counted per experiment. (**C**) ChIP-qPCR results showing enrichment of APE1 and XPA to B-DNA or H-DNA reporters under conditions of OS. Biological replicate means are shown ± SD and represent the mean of at least three independent experiments; **P< .01.

To explore the mutagenic potential of H-DNA-forming sequences in the presence of oxidative DNA damage, undamaged, or damaged mutation reporters containing H-DNA- or B-DNA-forming sequences were transfected into mammalian cells as previously described [17]. The spontaneous H-DNA-induced mutation frequency in the absence of OS yielded a seven-fold increase compared to control B-DNA (Fig. 5A), consistent with our previous results [17]. As expected, the overall mutation frequencies increase in both H-DNA and control B-DNA regions with increased OS with H-DNA being more mutagenic than B-DNA at low levels of OS. Under these conditions, the H-DNA substrate exhibited a three-fold higher mutation frequency compared to the oxidatively damaged B-DNA template. Overall, the fold increase in H-DNA-induced mutation frequency compared to B-DNA was substantially attenuated such that the H-DNA- and B-DNA-induced mutation frequencies were similar at the highest levels of OS (Fig. 5A).

Next, we compared the mutation spectra in mammalian cells at different levels of OS. Analyses reveal that without OS, the mutations obtained from B-DNA primarily consist of point mutations while H-DNA contains point mutations and large deletions, the latter which is a characteristic mutation type induced by H-DNA (Fig. 5B) [17]. As OS increases, the mutation spectra for H-DNA (mostly large deletions) transition to primarily point mutations, a signature mutation resulting from oxidative damage among canonical B-DNA structures [49, 50]. This suggests that the oxidized H-DNA-forming sequences are processed differently than H-DNA-forming sequences without oxidative damage in mammalian cells. Further, the shift in the signature mutation profile from large deletions (H-DNA generated) to point mutations (B-DNA generated) along with the attenuation in the fold increase in H-DNA-induced mutation frequency at increased OS suggest that the mutagenic processing mechanism of H-DNA becomes altered at higher levels of OS.

Approximately 28%, 25%, and 12.5% of the total mutations occur from large deletions as OS increases between the three treatment groups in the H-DNA reporter (Fig. 5B). This decrease in the percentage of large deletions is associated with a concomitant increase in the percentage of point mutations (Fig. 5B). Although the percentage of large deletions decreases due to the substantial increase in the overall mutation frequency from 18.5 × 10^−4^ to 247 × 10^−4^, the total number of large deletions remains the same among the varying levels of damage within the H-DNA reporters. Specifically, without OS, the H-DNA reporter has 5 large deletions per 10,000 total mutants but increases to ∼30 large deletions per 10,000 mutants for the H-DNA reporter at both levels of OS (Fig. 5B). Hence, H-DNA retains its characteristic mutation spectra, mostly of large deletions, at all levels of oxidation. This suggests that the mechanism responsible (e.g. NER) [23] for the formation of large deletions in the damaged H-DNA is independent of OS levels. This also suggests that the substantial increase in point mutations likely arises from a separate repair mechanism such as BER, which repairs oxidative DNA lesions induced by OS. Altogether, the accumulation of 8-oxodG within oxidatively damaged H-DNA (Fig. 2) is consistent with the accumulation of signature point mutations associated with OS (Fig. 5B).

### Differential recruitment of DNA repair proteins, APE1 and XPA, to oxidatively damaged H-DNA

The presence of oxidative lesions within the H-DNA-forming sequence and/or the destabilization of the H-DNA-forming substrate resulting from oxidative damage may affect the recruitment of DNA repair proteins to damaged sites within H-DNA-forming sequences. Although oxidative DNA lesions within B-DNA are primarily repaired by the BER pathway, H-DNA-forming sequences are primarily processed by the NER mechanism [23]. However, it is not known which pathway is involved in the repair of H-DNA-forming sequences containing oxidative damage. Thus, we examined the association of a key DNA repair protein from NER (XPA), and one from BER (APE1) with oxidatively damaged H-DNA-forming sequences. In the BER pathway, removal of an 8-oxoG base by 8-oxoguanine DNA glycosylase (OGG1) generates an abasic site which is then cleaved by the apurinic/apyrimidinic endonuclease 1 (APE1) to generate a nick. In the NER pathway, xeroderma pigmentosum complementation group A (XPA) is a key scaffold/verification protein recruited to a broad range of helix-destabilizing lesions and has been shown to act on H-DNA substrates [23].

Here, we performed ChIP assays to determine the association of APE1 and XPA repair proteins to B-DNA and H-DNA-forming sequences in the presence or absence of oxidative damage in mammalian cells. As expected, there is a statistically significant enrichment of APE1 to the control B-DNA template with increased OS with an approximately eight-fold increase in APE1 recruitment at higher levels of OS over the undamaged B-DNA sequence (Fig. 5C). In contrast, the association of XPA with B-DNA does not increase upon OS. Our ChIP results also confirm that XPA is recruited to undamaged H-DNA, consistent with our previous results (Fig. 5C) [23]. Interestingly, we observe the recruitment of APE1 to the undamaged H-DNA-forming sequence suggesting an involvement of this BER protein in recognizing/processing H-DNA. At lower levels of OS, APE1 remains associated with the damaged H-DNA-forming sequence. However, at higher levels of OS, APE1 recruitment significantly decreases by almost half when compared to the undamaged H-DNA-forming sequence. Conversely, XPA recruitment significantly accumulates with increased OS, resulting in an approximately four-fold increase at the higher level of OS compared to the undamaged H-DNA-forming sequence. Thus, we found that increasing OS triggers an enhanced association of XPA to oxidatively damaged, H-DNA-forming sequences, but not in B-DNA. Based on these results, proteins from both the BER and NER pathways have significant alterations in their association with B-DNA- and H-DNA-forming sequences depending on the level of OS.

## Discussion

Oxidative DNA damage is a major threat to the integrity of the genome and can be caused by a variety of sources that generate intracellular ROS including aerobic metabolism. Under conditions of OS, elevated levels of ROS have been implicated in the etiology of cancer. Of particular interest, H-DNA-forming sequences, which are enriched at mutation hotspots in human cancer genomes, have emerged as an endogenous source of genetic instability and have also been implicated in cancer etiology. Several types of non-B DNA, with their structural alterations and richness in guanines, may be more vulnerable to DNA damage as guanines have the lowest redox potential of the four DNA bases [25]. Our central question in this study explores the effect of oxidative DNA damage within H-DNA-forming sequences on its stability and mutagenic potential. Here, we characterize the effects of oxidative damage on H-DNA structure formation as well as H-DNA-induced mutagenesis, thus providing a mechanistic framework that links OS levels to the altered biological processing of H-DNA and enhanced genetic instability.

Our results demonstrate, for the first time, that oxidative DNA damage accumulates at H-DNA-forming sequences likely attributed to structural alterations associated with H-DNA. H-DNA has higher background levels of endogenous DNA damage, likely produced during the isolation process (Figs 2B and 3). Upon OS, strand-specific oxidized purine and pyrimidine bases are induced within the H-DNA-forming sequences. Specifically, oxidized purines accumulate within the R-triplex strand of the H-DNA-forming sequences, compared to control B-DNA sequences with the same GC content (Fig. 3). Furthermore, there was a pronounced accumulation of oxidized pyrimidine bases within the Y-loop strand of the H-DNA structure (Fig. 3A) that was induced by OS whereas there was no significant increase in oxidized pyrimidines within the equivalent strand in the B-DNA samples, suggesting that the single-stranded region of H-DNA is a hotspot for oxidative DNA damage. Single-stranded DNA has been reported to be more sensitive to DNA damage and mutations than double-stranded DNA [34]. Furthermore, guanine oxidation products, rates of oxidation, and location of oxidation are influenced by secondary structure [51, 52]. In fact we have identified significant genome-wide colocalization between oxidative lesions and H-DNA-forming sequences [40]. Hence, the unique structural features of H-DNA may increase its susceptibility to oxidative damage, rendering it a molecular target for oxidative damage. As a result, an accumulation of damage within H-DNA-forming regions occurs that subsequently amplifies the biological impact of H-DNA and contributes to enhanced mutagenesis (Figs 2 and 3).

Oxidative damage can influence H-DNA structure formation, thereby modulating DNA damage recognition and the mutagenic processing of H-DNA. Therefore, we examined how oxidative DNA lesions impact H-DNA conformation and stability. Examination of the CD spectra revealed that the conformations of the oxidative lesion-modified R2 substrates are similar to the unmodified R2 (Fig. 4C) and that the presence of a single 8-oxodG or abasic site did not result in any significant structural or conformational changes. However, based on our gel electrophoresis experiments, the presence of an abasic site at some locations (AB15 and AB30) results in the formation of a less compact structure compared to the unmodified R2 (Fig. 4B). On the other hand, our thermal melting experiments indicate that the incorporation of a single 8-oxodG or an abasic site destabilizes the H-DNA structure, likely due to alterations in structural flexibility (Fig. 4D). The extent of destabilization depends on the type of lesion and its location. 8-oxodG within the Watson–Crick duplex (OG15 and OG19) destabilizes the H-DNA structure to a greater extent than one positioned within the reverse Hoogsteen triplex region (OG30), with a T_m_ depression of ∼6°C and 4°C, respectively, while an abasic site within the Watson–Crick duplex and the reverse Hoogsteen triplex yields a T_m_ depression of around 15°C and 7°C, respectively. Comparatively, previous work by the Breslauer group reported that 8-oxodG within a duplex decreases the T_m_ by ∼3°C while an abasic site decreases the T_m_ by almost 14°C relative to the unmodified B-DNA duplex [53, 54]. The oxygen atom at the 8th position in 8-oxodG does not disrupt Watson–Crick hydrogen bonding interactions with the opposite cytosine in B-DNA duplexes, but enhances flexibility in duplex B-DNA [55]. It is possible that our observed decrease in T_m_ at positions 15 and 19 could be attributed to local conformational changes within the H-DNA structure. On the reverse Hoogsteen triplex strand, the presence of 8-oxodG at position 30 (OG30) could potentially disturb the reverse Hoogsteen H-bond arrangement resulting in reduced thermal stability. Abasic site modification further destabilizes the H-DNA structure due to the absence of a base that contributes to base stacking and stabilization. Indeed, oxidative lesions such as 8-oxodG were previously shown to impact the stability and structure of several non-B DNA structures, including G4-DNA, hairpins, and Z-DNA [39,56–61]. While our analysis only involved the impact of a single lesion, numerous lesions are likely to have a greater impact on the structure and stability of H-DNA. Further characterization is needed to identify specific structural alterations and perturbations as a result of oxidative damage within H-DNA.

We next sought to investigate if the accumulation of oxidative lesions within H-DNA impacts its mutagenic potential by measuring the mutation frequencies and spectra for control B-DNA or H-DNA-forming sequences exposed to OS. As expected, OS elevates the mutation frequency of both sequences (Fig. 5A). Multiple lesions within B-DNA-forming sequences can enhance their mutagenetic potential [62], which may explain the general increase in mutation frequencies at higher levels of OS within B-DNA and H-DNA-forming sequences in our study. However, H-DNA has a higher mutation frequency than B-DNA at undamaged and lower OS levels, indicating that H-DNA is more mutagenic than B-DNA as we have previously shown [17]. Interestingly, the fold difference in mutation frequencies between B-DNA and H-DNA decreases with increased OS, demonstrating that the mutagenic potential of H-DNA is attenuated under these conditions. One interpretation of this observation is that B-DNA and H-DNA-forming sequences may be saturated with DNA damage at higher levels of OS. Another possibility is that high levels of oxidative damage disrupt the H-DNA conformation such that oxidative damage within B-DNA and H-DNA may be processed in a similar fashion. However, the observed mutation spectra between the B-DNA and H-DNA-forming sequences do not fully support this idea as they are not identical. Oxidatively damaged H-DNA retains its characteristic mutagenic signature (i.e. large deletions) that are not detected in the B-DNA samples (Fig. 5B). Thus, our results provide evidence that the accrued oxidative damage within H-DNA-forming sequences accounts for the observed alterations in H-DNA-induced mutagenesis. Furthermore, the mutation spectra of oxidatively damaged H-DNA consist of a combination of point mutations and large deletions characteristic of repair processing of B-DNA containing oxidative base lesions and H-DNA, respectively. This suggests that dual repair pathways are likely involved in the processing of oxidatively damaged H-DNA.

The results from the mutation spectra prompted us to investigate the role of DNA repair proteins that may associate with oxidized H-DNA sequences. Our ChIP results reveal that APE1 and XPA are enriched at both undamaged and oxidatively damaged H-DNA, suggesting that the BER and NER pathways may both be involved in the repair process (Fig. 5C). BER is the major mechanism involved in processing oxidized base lesions within B-DNA while NER primarily processes damage that distorts the DNA helix, including H-DNA [23]. Although XPA recruitment to undamaged H-DNA was expected from our previously published studies [23], the discovery that APE1 is also recruited to undamaged H-DNA is unexpected. One possible explanation is that the enhanced background levels of oxidative lesions present in undamaged H-DNA compared to undamaged B-DNA (Figs 2 and 3) facilitates APE1 recruitment. APE1 has also been shown to remove oxidized base lesions in single-stranded DNA and hairpin loops of trinucleotide repeats [62, 63] and could thus potentially recognize oxidized base lesions within H-DNA. Second, recent evidence suggests that NER and BER pathways compete to mediate the removal of oxidized guanine lesions within B-DNA, implicating NER proteins in the repair of oxidative damage [64]. This could explain the recruitment of both APE1 and XPA to undamaged H-DNA because the two repair proteins are known to recognize oxidized base lesions inherently present in H-DNA (Fig. 5C). In any case, our results demonstrate an enrichment of APE1 and XPA at the undamaged H-DNA region when compared to the undamaged B-DNA control region.

Interestingly, increased levels of OS caused a marked shift in the mutation spectra and the greatest impact on the loss of fold induction of mutations with H-DNA over that of B-DNA. Specifically, there is a shift where XPA association with H-DNA is enhanced significantly and APE1 association is reduced at higher levels of OS (Fig. 5C). In other words, higher levels of oxidative DNA damage promote the specific recruitment of XPA to H-DNA. Oxidized lesions may perturb the H-DNA structure and alter the binding energetics of the protein–DNA interactions. This could explain the enhanced association of XPA, which is known to display specificity for structurally distorted DNA and recognize single-stranded branches and bubble substrates with high affinity [65]. Alternatively, the dynamic equilibrium between the formation of H-DNA and B-DNA containing oxidative damage could modulate the binding affinity of repair proteins, where XPA is recruited to the H-DNA structure and APE1 recognizes lesions within B-DNA. Importantly, despite this robust enrichment of XPA at H-DNA at higher levels of OS; surprisingly, the relative frequency of large deletions did not increase (Fig. 5B). This suggests that XPA, although enriched at H-DNA, did not process the oxidatively damaged H-DNA into large deletions as we previously observed with undamaged H-DNA [17]. Strikingly, as the number of point mutations increases (Fig. 5B), the recruitment of APE1 decreases (Fig. 5C) in H-DNA at higher levels of OS, suggesting that other proteins/pathways that generate point mutations most likely play a role. We surmise that although there was increased enrichment of XPA to a structurally distorted H-DNA conformation containing higher levels of oxidative damage, NER may not have been able to process this structure as it would for an undamaged H-DNA structure, and instead relies upon other proteins/pathways for its processing. Further studies are warranted to determine the detailed mechanism(s) of oxidatively damaged H-DNA processing.

**Scheme 1.**
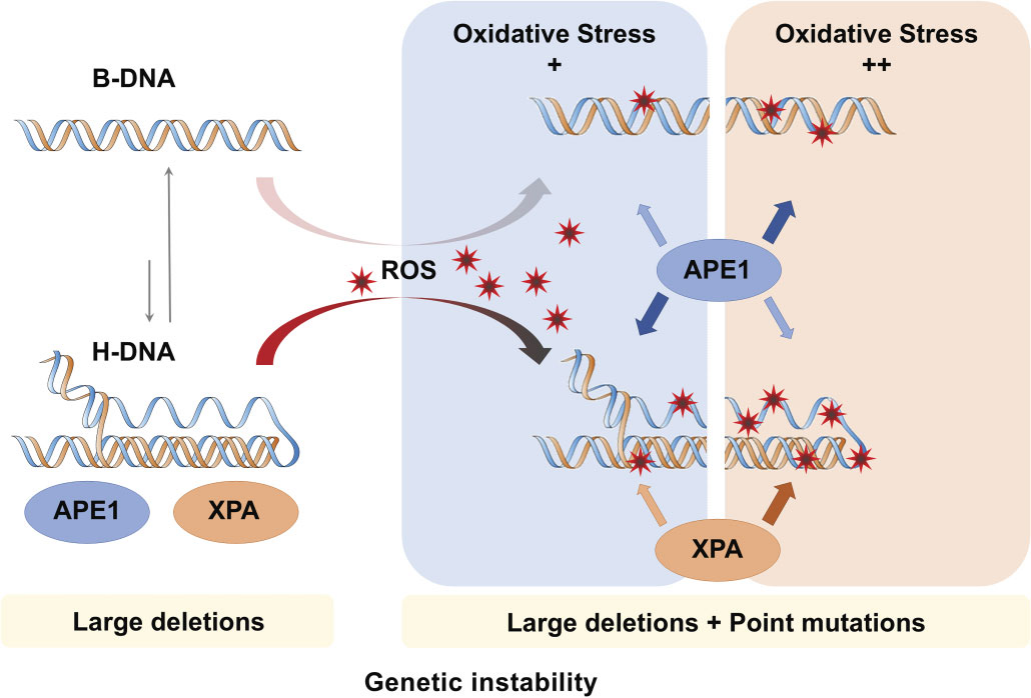
Schematic diagram illustrating the association of APE1 and XPA with B-DNA- and H-DNA-forming sequences depending on the extent of OS. Our previous results demonstrate that H-DNA is processed in a mutagenic fashion by the NER mechanism, leading predominantly to large deletions [23]. Without OS (left), both APE1 and XPA are recruited to H-DNA. Under OS, H-DNA accumulates ROS-induced oxidative damage in the pyrimidine-rich loop and the purine-rich triplex, which results in the destabilization of the H-DNA structure. This increase in OS leads to the preferential recruitment of APE1 or XPA to H-DNA (right). Mutagenic DNA repair processing shifts from primarily large deletions in undamaged H-DNA to an accumulation of large deletions and point mutations in oxidatively damaged H-DNA.

Taken together, we propose the following model outlining the impact of oxidative damage on H-DNA-induced mutagenesis (Scheme 1). In the absence of OS, H-DNA-forming sequences yield large deletions through NER processing in mammalian cells [23]. However, under OS, H-DNA-forming sequences accumulate more oxidative damage compared to B-DNA, which destabilizes the H-DNA structure. As a result, mutagenic DNA repair processing shifts from primarily large deletions in undamaged H-DNA to an accumulation of point mutations in oxidatively damaged H-DNA. Elevated levels of oxidative damage result in the differential association of repair proteins from both the BER and NER pathways that is unique with oxidatively damaged H-DNA and not B-DNA. This suggests that both APE1 and XPA are able to recognize and respond to H-DNA containing oxidative damage. It is intriguing to speculate that some other repair pathway may be involved in the mutagenic processing that is responsible for the higher levels of point mutations.

In summary, H-DNA contributes to genetic instability through multiple mechanisms with distinct mutagenic outcomes that depend on the extent of OS. We have previously established that H-DNA-forming sequences co-localize with mutation hotspots and are an endogenous source of instability [17,20,23]. Our results here demonstrate that H-DNA-forming regions are hotspots for oxidative damage and subsequent mutagenesis, resulting in aberrant DNA repair processing of H-DNA and increased genetic instability. These results suggest that OS commonly found in tumor microenvironments elevates H-DNA-induced genetic instability and informs how mutation hotspots are generated. Together, these results reveal a new model of genetic instability that links DNA structure and OS to cancer etiology and other genetic diseases, and helps to establish alternative DNA-forming sequences as a promising genomic biomarker and therapeutic target in genetic diseases, such as cancer.

## Supplementary Material

gkaf066_Supplemental_File

## Data Availability

Experimental data are available upon reasonable request by writing to the corresponding author.
