# Supplementary material for: Oxidative damage within alternative DNA structures results in aberrant mutagenic processing
Source: Nucleic Acids Res. 2025 Feb 14;53(4):gkaf066. doi: 10.1093/nar/gkaf066 (PMC11826088; doi:10.1093/nar/gkaf066)

Supporting Information

Oxidative damage within alternative DNA structures results in aberrant mutagenic processing.

Maha Zewail-Foote[1], Imee M.A. del Mundo[2], Alex W. Klattenhoff[2], Karen M. Vasquez*[2]

[1]Department of Chemistry and Biochemistry, Southwestern University, 1001 E University Ave, Georgetown, TX, USA

[2]Department of Pharmacology and Toxicology, College of Pharmacy, The University of Texas at Austin, Dell Pediatric Research Institute, 1400 Barbara Jordan Blvd. Austin, TX, USA.

*To whom correspondence should be addressed: Tel: +1 512 495 3040; Fax: +1 512 495 4946; Email: karen.vasquez@austin.utexas.edu

Figure S1. Oligonucleotides used in this study. The R2 sequence is derived from a chromosomal breakpoint hotspot in the human c-MYC gene and previously characterized.[1] Specific guanine bases (magenta) were replaced with either 8-oxodG (OG) or an abasic site (AB) based on the numbering scheme shown in the 36-bp H-DNA substrate (right). Guanine substitutions occurred either in the Watson-Crick duplex region (underlined, positions 15 and 19), the single-stranded loop (position 23), or within the triplex reverse Hoogsteen strand (red, position 30); *Reverse Hoogsteen hydrogen bonds.

Name	Sequence (5’ ® 3’)
R2	CCCCTCCCTTTTTGGGAGGGGCGCTTATGGGGAGGG
OG/AP15	CCCCTCCCTTTTTG**G**GAGGGGCGCTTATGGGGAGGG
OG/AP19	CCCCTCCCTTTTTGGGAG**G**GGCGCTTATGGGGAGGG
OG/AP23	CCCCTCCCTTTTTGGGAGGGGC**G**CTTATGGGGAGGG
OG/AP30	CCCCTCCCTTTTTGGGAGGGGCGCTTATG**G**GGAGGG

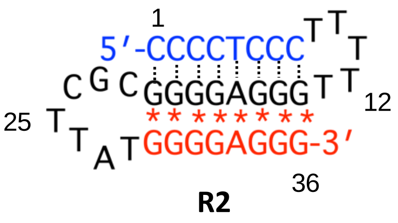

Supplementary references

1. Del Mundo, I. M. A., Zewail-Foote, M., Kerwin, S. M. & Vasquez, K. M. Alternative DNA structure formation in the mutagenic human c-MYC promoter. *Nucleic Acids Res* **45**, 4929-4943 (2017).

Figure S2. S1 nuclease mapping. The formation of H-DNA in the two plasmid constructs used in this study was verified using S1 nuclease with restriction enzyme (RE) mapping for each plasmid construct (A, B). The formation of H-DNA is confirmed by the appearance of specific cleavage products (indicated by arrows) in lanes 8, corresponding to the predicted single-stranded regions and their positions relative to the restriction sites. The molecular weight marker (MM) is included for size reference.

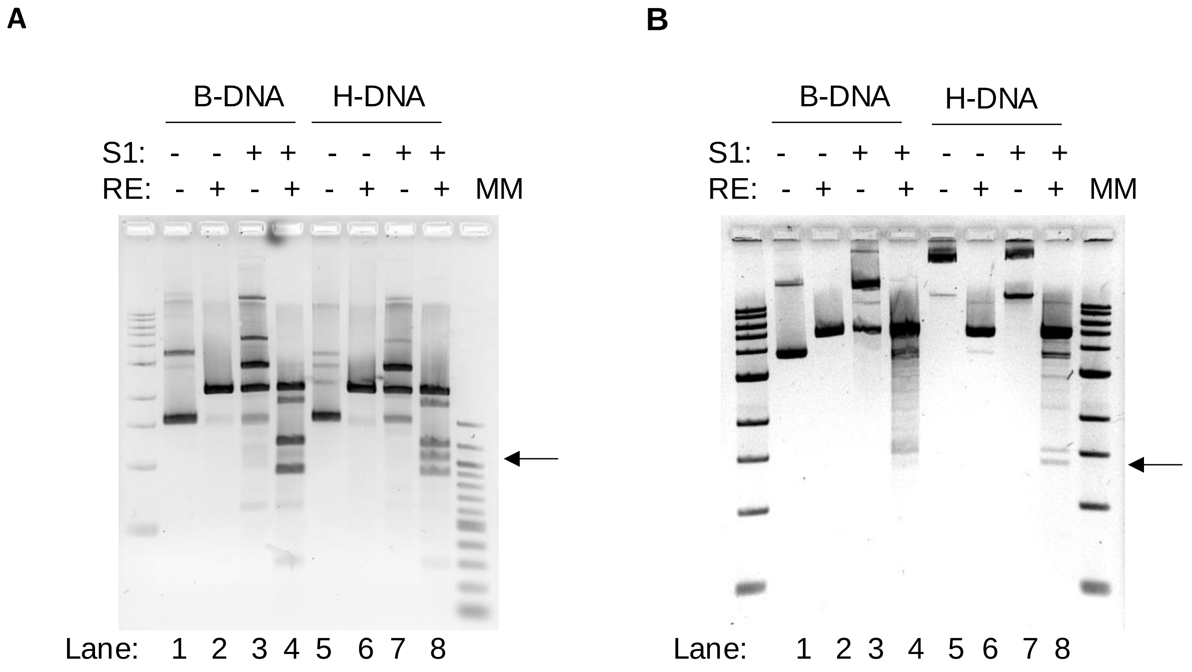

Supplement: gkaf066_Supplemental_File [file gkaf066_supplemental_file.pdf]
